# 

**Published:** 1907-09

**Authors:** 


					CONTENTS.
Page
Rheumatic Carditis in Childhood.
By Carey Coombs, M.D. Lond.
193
Treatment of Graves's Disease by Anti-Thyreoid Serum and by
X-Rays.
By J. Michell Clarke, M.A., M.D. Cantab., F.R.C.P.
Cerebral Lesions in Pregnancy and Parturition.
By Walter C.
Swayne, M.D , B.S. Lond.
209
A Suggested Treatment for Functional Aphonia.
By C. Percival
Crouch, M.B. Lond., F.R.C.S.
214
A Case of Filariasis : Removal of Lymphatic Varix by Operation.
By J. Paul Bush, C.M G., and J. A. Nixon, M.B. Cantab., M.R.C.P.
218
The Medical Reading Society, Bristol.
By L. M. Griffiths
Progress of the Medical Sciences
Medicine.
I. Walker Hall, M.D. Vict.
236
Surgery.
T. Carwakdine, M.B., M.S. Lond.
243
Ophthalmology.
A. Ogilvy, M.D., B. Ch., B.A. Dub.
247
Reviews of Books
251
Editorial Notes
272
Notes on Preparations for the Sick
278
The Library of the Bristol Medico-Chirurgical Society   283
Meetings of Societies
285
Local Medical Notes
287

				

